# How Transportation Restriction Shapes the Relationship Between Ambient Nitrogen Dioxide and COVID-19 Transmissibility: An Exploratory Analysis

**DOI:** 10.3389/fpubh.2021.697491

**Published:** 2021-07-29

**Authors:** Lefei Han, Shi Zhao, Peihua Cao, Marc K. C. Chong, Jingxuan Wang, Daihai He, Xiaobei Deng, Jinjun Ran

**Affiliations:** ^1^School of Global Health, Chinese Center for Tropical Diseases Research, Shanghai Jiao Tong University School of Medicine, Shanghai, China; ^2^The Jockey Club (JC) School of Public Health and Primary Care, Chinese University of Hong Kong, Hong Kong, China; ^3^The Chinese University of Hong Kong (CUHK) Shenzhen Research Institute, Shenzhen, China; ^4^Clinical Research Center, Zhujiang Hospital, Southern Medical University, Guangzhou, China; ^5^Department of Applied Mathematics, Hong Kong Polytechnic University, Hong Kong, China; ^6^School of Public Health, Shanghai Jiao Tong University School of Medicine, Shanghai, China

**Keywords:** COVID-19, nitrogen dioxide, reproduction number, transportation, China

## Abstract

**Background:** Several recent studies reported a positive (statistical) association between ambient nitrogen dioxide (NO_2_) and COVID-19 transmissibility. However, considering the intensive transportation restriction due to lockdown measures that would lead to declines in both ambient NO_2_ concentration and COVID-19 spread, the crude or insufficiently adjusted associations between NO_2_ and COVID-19 transmissibility might be confounded. This study aimed to investigate whether transportation restriction confounded, mediated, or modified the association between ambient NO_2_ and COVID-19 transmissibility.

**Methods:** The time-varying reproduction number (*R*_*t*_) was calculated to quantify the instantaneous COVID-19 transmissibility in 31 Chinese cities from January 1, 2020, to February 29, 2020. For each city, we evaluated the relationships between ambient NO_2_, transportation restriction, and COVID-19 transmission under three scenarios, including simple linear regression, mediation analysis, and adjusting transportation restriction as a confounder. The statistical significance (*p*-value < 0.05) of the three scenarios in 31 cities was summarized.

**Results:** We repeated the crude correlational analysis, and also found the significantly positive association between NO_2_ and COVID-19 transmissibility. We found that little evidence supported NO_2_ as a mediator between transportation restriction and COVID-19 transmissibility. The association between NO_2_ and COVID-19 transmissibility appears less likely after adjusting the effects of transportation restriction.

**Conclusions:** Our findings suggest that the crude association between NO_2_ and COVID-19 transmissibility is likely confounded by the transportation restriction in the early COVID-19 outbreak. After adjusting the confounders, the association between NO_2_ and COVID-19 transmissibility appears unlikely. Further studies are warranted to validate the findings in other regions.

## Introduction

Since the coronavirus disease 2019 (COVID-19) was first reported in December 2019 in China, the cumulative cases and death cases, as of May 2021, have been over 160 million and 3.4 million, respectively (1). In response to the rapid transmission of COVID-19, many authorities enforced lockdown measures regionally aiming to restrict the social contact and limit the virus transmission to reduce the morbidity and mortality caused by COVID-19 (2). In China, intensive non-pharmaceutical interventions, including city lockdown measures, have been implemented at both the provincial and city levels about three weeks after the first cases were reported, i.e., by the end of January 2020.

Recent evidence shows that the city lockdown measures, especially for transportation restriction, have resulted in a reduction in the levels of air pollution, including nitrogen dioxide (NO_2_) (3–5). Ambient NO_2_ is mainly generated from fossil fuels burning through automobile exhaust and industrial emissions. Several studies indicate that NO_2_ positively associates with the COVID-19 transmissibility (6–9), though results were not always consistent (10, 11). An experimental study found NO_2_ exposure increased the expression of angiotensin-converting enzyme 2 (ACE2), which might lead to increased susceptibility to virus infections (12, 13). Exposing to a higher concentration of NO_2_ also lead to respiratory functionality damage, including decreased levels in lung volume and expiratory flow (14). Given that the impact of transportation restriction on ambient NO_2_ and COVID-19 transmissibility have been well understood (15, 16), we speculate that the statistical association between ambient NO_2_ and COVID-19 transmissibility obtained from previous evidences may be undermined without considering the effect of transportation restriction.

This study aimed to explore whether transportation restriction during the implementation of COVID-19 lockdown measures would modify the association between ambient NO_2_ on COVID-19 transmissibility in different scenarios.

## Methods

### Data

Daily counts of cumulative COVID-19 deaths for each Chinese city were obtained from the China National Health Commission and the Chinese provincial health agencies. Cities with cumulative cases over 100 on February 5, 2020 were included in our analysis. The study period was set from January 1, 2020 to February 29, 2020. Daily mean concentrations of NO_2_ during the same period were obtained from the China National Environmental Center. Information on the date and control measures of ‘the first-level response', i.e., when the transportation restriction was implemented, was collected from the government website or official media of each province. We set a time-varying binary variable (i.e., 0 and 1) before and after the date of lockdown for each city.

### COVID-19 Transmissibility

We adopted the time-varying reproduction number (*R*_*t*_) to quantify the instantaneous COVID-19 transmissibility in each Chinese city (17). Following the estimation framework developed in previous studies (17–19), the epidemic growth of COVID-19 was modeled as a branching process, and thus, *R*_*t*_ can be expressed by using the renewable equation as follows:

$$\begin{array}{l} R\left(t\right)=\frac{C\left(t\right)}{\int_{0}^{\infty }w\left(k\right)C\left(t-k\;\right)dk}, \end{array}$$

where *C*(*t*) is the number of COVID-19 cases at the *t*-th date. The function *w*(·) is the distribution of the generation time (GT) of COVID-19. By averaging the GT estimates from the existing literature (20–24), we considered *w* as the Gamma distribution with a mean (±SD) value of 5.3 (±2.1) days. Slight variations in the settings of the GT did not affect our main findings.

### Statistical Analysis

**Figure 1:**
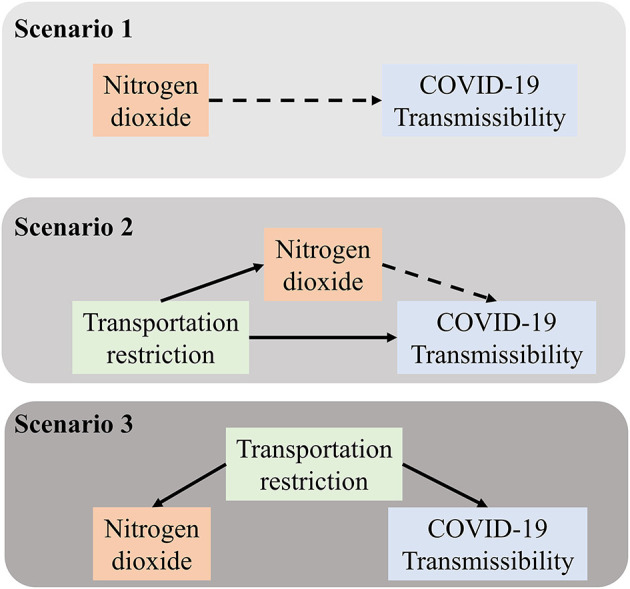
Directed acyclic graphs of Scenario 1, Scenario 2, and Scenario 3. Scenario 1 shows the possible association between NO_2_ and COVID-19 transmissibility; Scenario 2 shows the direct association between transportation restriction and COVID-19 transmissibility as well as the indirect association mediated by NO_2_; and Scenario 3 shows that the possible confounding of transportation restriction on the association between NO_2_ and COVID-19 transmissibility.

We explored the role of ambient NO_2_ in affecting the *R*(*t*) with three different scenarios. They included the following (Figure 1): Scenario 1: simple linear regression (naïve scenario); Scenario 2: mediation analysis; and Scenario 3: adjusting for confounding.

#### Scenario 1: Simple Linear Regression

The associations between air pollutants and epidemiological outcomes at population scale are commonly explored by using regression models, which link the two terms directly in one formula with or without adjusting other common covariables (25). The ambient NO_2_ is found positively associated with the COVID-19 transmissibility in recent literature (6). As for a start-up, we repeatedly adopted the simple linear regression models and reproduced the positive association between NO_2_ and COVID-19 transmissibility using the dataset in this study. We attempted three schemes to quantify this association, including: (i) univariate regression; (ii) multivariate regression with temperature and relative humidity adjusted (26); and (iii) Pearson and Spearman ranked correlations. To be consistent with previous findings, a positive and significant association between ambient NO_2_ and COVID-19 transmissibility was desired.

#### Scenario 2: A Mediation Analysis

In the hypothesized mediation framework, we considered transportation restriction, ambient NO_2_, and COVID-19 transmissibility as the independent variable, mediator, and the dependent variable, respectively. The assumption is based on the well-studied evidence that (i) transportation restriction causes a reduction in ambient NO_2_ (3) and (ii) transportation restriction may also reduce the transmissibility of COVID-19 (15, 16). According to the mediation framework by the classic requirements of Baron and Kennys (27), NO_2_ would be a mediator to explain the relationship between transportation restriction and COVID-19 transmissibility if the hypothesis yielded in Scenario 1 was true.

We examined the mediation effects by two measurements, which are as follows: (i) absolute mediation effect and (ii) proportional mediation effect. If there exists an association between ambient NO_2_ and COVID-19 transmissibility, the direct association between transportation restriction and COVID-19 after considering ambient NO_2_ (indirect association) is expected to reduce. Otherwise, the association yielded in Scenario 1 is suspicious and unlikely to imply causality, but merely reflects the relationship caused by transportation restriction.

#### Scenario 3: Adjusting Transportation Restriction as A Confounder

In the situation that the mediation effect is not of statistical significance, we suspect that transportation restriction might confound the relation yielded in Scenario 1. We adopted the two regression models to examine the adjusted association between ambient NO_2_ and they are as follows: (i) multivariate regression with transportation restriction adjusted and (ii) multivariate regression with transportation restriction, temperature, and relative humidity adjusted. Here, the adjusted association indicates an impact on COVID-19 transmissibility that is solely contributed by NO_2_.

The three different analytical scenarios are nested progressively. Specifically, the estimating outcomes in Scenario 1 serve as the presumption of the modeling framework in Scenario 2. The estimating outcomes in Scenario 2 may support the intuition of the formulation in Scenario 3.

We conducted statistical analysis across 31 selected cities, and obtained the city-level statistical significance (*p*-value) in three different scenarios. For regression models, *p*-values are calculated by using the Student's *t*-test. For mediation analysis and non-parametric statistics, *p*-values are calculated by using bootstrapping sampling with 1,000 runs of the simulation. All tests are one-sided. A *p*-value <0.05 is considered as statistical significance. We summarized the percentage distribution of all statistically significant *p*-values across all the 31 selected cities yielded from our models for comparison.

All analyses were carried out using **R** statistical program language (version 3.6.0) (28).

## Results

Of the 31 selected Chinese cities included in our analysis, 13 cities were from Hubei province and 18 cities from other regions. The date of lockdown intervention in the included cities was distributed from January 23, 2020 (e.g., Wuhan) to January 27, 2020 (e.g., Shenzhen). The ambient average concentration of NO_2_ ranged from 14.0 μg/m^3^ (Enshi) to 47.3 μg/m^3^ (Tianjin) during the study period.

The percentage distribution of *p*-values on the association between NO_2_ and COVID-19 transmissibility by different measurements across the 31 selected cities are summarized in Table 1. In Scenario 1, 77.4–87.1% cities show that the relationship between NO_2_ and COVID-transmissibility reached statistical significance (*p*-value < 0.05) with regards to either Pearson, Spearman correlation coefficients or regression coefficients. In Scenario 2 where NO_2_ is treated as a mediator between transportation restriction and COVID-19 transmissibility, we find that the *p*-value of either absolute effect or proportional effect lost statistical significance in most of the cities. In Scenario 3 where transportation restriction is treated as a confounder in the regression model, little evidence about the association between NO_2_ and COVID-19 transmissibility is observed.

**Table 1:** Summary of *p*-values of all *N* = 31 selected cities, and comparison of the percentage distribution of *p*-values by different measurements across the 31 cities.

**Framework**		**Measurement**	**Covariable adjustment**	***p*** **-values (of** ***N*** **=** **31 cities)**			**Prop. of *p*-value < 0.05(out of *N* = 31 cities)**
				**First-quarter**	**Median**	**Third-quarter**	
Scenario 1	Correlation	Pearson coefficient	NA	<0.001	0.003	0.012	83.9%
		Spearman coefficient	NA	<0.001	0.002	0.027	87.1%
	Naïve regression	Regression coefficient	No	<0.001	0.003	0.014	83.9%
			Yes	<0.001	0.006	0.028	77.4%
Scenario 2	Mediation effect	Absolute effect	No	0.051	0.282	0.496	22.6%
			Yes	0.060	0.210	0.387	16.1%
		Proportional effect	No	0.072	0.324	0.580	12.9%
			Yes	0.174	0.288	0.621	6.5%
Scenario 3	Confounder adjustment	Regression coefficient	No	0.121	0.312	0.653	3.2%
			Yes	0.201	0.408	0.625	6.5%

*Covariable adjustment, adjusting for temperature and relative humidity*.

*Scenario 2, setting NO_2_ concentration as the mediator of indirect effect from the transportation restriction to the decline of COVID-19 transmissibility*.

*Scenario 3, setting the transportation restriction as the confounder of NO_2_ concentration and COVID-19 transmissibility*.

*For regression models, p-values were calculated by Student's t-test*.

*For mediation analysis and non-parametric statistics, p-values were calculated by bootstrapping sampling with 1,000 runs of the simulation*.

## Discussion

This study evaluated the association between NO_2_ and COVID-19 transmissibility with and without considering the impact of transportation restriction in the three different scenarios. Our results did not support that NO_2_ was a mediator between transportation restriction and COVID-19 transmissibility. We did not observe that NO_2_ was independently associated with COVID-19 transmissibility after adjusted for transportation restriction either.

Our study adopted three hypothesis scenarios to evaluate the association of NO_2_ and COVID-19 transmissibility by several statistical approaches in each scenario. The results were stable in both the analytic approaches and hypothesis framework. Instead of using the daily number of cases as the outcome, we adopted *R*_*t*_ to represent the disease transmissibility, which would avoid autocorrelation among cases and avoid over interpreting the association between environmental factors and COVID-19 (29).

Our result in Scenario 1 was consistent with the previous study in China (9). However, the statistical model used in the previous study was limited to the control of population movement and transportation restriction due to the data availability. An ecological study in Milan, Italy showed NO_2_ was inversely correlated with the total number of cases, daily new cases, and total deaths of COVID-19 infections (30). However, the impact of lockdown on NO_2_ and COVID-19 was not adequately evaluated. In addition, this study may also be restricted due to the use of aggregated number of COVID-19 cases for analysis (29).

Our results in Scenarios 2 and 3 showed that the association between ambient NO_2_ and COVID-19 transmissibility yielded in Scenario 1 might be spurious. We suggested that transportation restriction served as a confounder in this association. Despite earlier studies suggesting the adverse impact of NO_2_ and human susceptibility on respiratory illness, including impaired function in the immune system (12, 31) and respiratory system (14), we did not observe the association of ambient NO_2_ for COVID-19 transmissibility in China. One possible explanation would be the lack of indoor NO_2_ data. During the early outbreak period of China, which was also the period of the Spring festival, people were more likely to stay in household environments with closed windows on such cold days. The chance of being exposed to ambient NO_2_ might be less likely and its impact would be smaller than the impact of transportation restrictions.

Our study has some limitations. First, the levels of transportation restriction across provinces within China were varied. For example, in the epicenter of Wuhan, Hubei Province, rigorous transportation restriction was implemented that prohibited all inter and intracity transport. In other cities out of Hubei Province, border shutdown, restriction of intercity travel, and intracity activities were implemented (32). The variated measures of transportation restriction made the data to be quantified challenging. We hence adopted a binomial variable of lockdown to represent transportation restriction in the analysis. Second, unmeasured factors, such as population density, flow, and local economy levels, are potential confounders which may be associated with transportation restriction and *R*_0_ (33, 34). Our results showed the *p*-values from Scenarios 2 and 3 were less likely to be smaller than statistical levels across cities, suggesting the impacts from unmeasured factors would not change our primary conclusion. Third, since the outbreak of COVID-19 in China occurred in the Spring festival and authorities implemented lockdown measures at both provincial and city levels, our results might not be generalizable to other regions that may have different lockdown measures. The extreme event that occurred in other countries, which might have an influence on NO_2_ concentration, transportation restriction, and COVID-19 spread, should also be considered (35). Further studies are warranted to test our findings.

In summary, we find little evidence about the association between ambient NO_2_ and COVID-19 transmissibility in China. Timely transportation restriction effectively reduced the transmissibility of COVID-19 during the early outbreak period. Despite this, given the global pandemic of COVID-19, the impact of ambient NO_2_ is still necessary to be evaluated in other regions.

## Data Availability Statement

The original contributions presented in the study are included in the article/supplementary material, further inquiries can be directed to the corresponding author/s.

## Author Contributions

JR and SZ designed the study. LH and PC contributed research data. LH, SZ, and JR contributed to data analysis and manuscript writing. All authors contributed to supervision, manuscript revision, and gave final approval for publication.

## Author Disclaimer

The funding agencies had no role in the design and conduct of the study; collection, management, analysis, and interpretation of the data; preparation, review, or approval of the manuscript; or decision to submit the manuscript for publication.

## Conflict of Interest

The authors declare that the research was conducted in the absence of any commercial or financial relationships that could be construed as a potential conflict of interest.

## Publisher's Note

All claims expressed in this article are solely those of the authors and do not necessarily represent those of their affiliated organizations, or those of the publisher, the editors and the reviewers. Any product that may be evaluated in this article, or claim that may be made by its manufacturer, is not guaranteed or endorsed by the publisher.
